# Epigenetic and blood markers associated with response to electroconvulsive therapy in patients with depressive disorders

**DOI:** 10.1038/s41398-025-03772-y

**Published:** 2025-12-03

**Authors:** Anne-Kristin Stavrum, Lea Sirignano, Leila M. Frid, Josef Frank, Jerome C. Foo, Leticia M. Spindola, Kira D. Höffler, Ketil J. Oedegaard, Jan Haavik, Marcella Rietschel, Stephanie H. Witt, Ute Kessler, Leif Oltedal, Stéphanie Le Hellard

**Affiliations:** 1 Department of Clinical Science, University of Bergen, Bergen, Norway; 2 Dr. Einar Martens Research Group for Biological Psychiatry, Centre for Medical Genetics and Molecular Medicine, Haukeland University Hospital, Bergen, Norway; 3 Bergen Center for Brain Plasticity, Haukeland University Hospital, Bergen, Norway; 4 Department of Genetic Epidemiology in Psychiatry, Central Institute of Mental Health, Medical Faculty Mannheim, University of Heidelberg, Mannheim, Germany; 5 Mohn Medical Imaging and Visualization Centre, Department of Radiology, Haukeland University Hospital, Bergen, Norway; 6 Department of Psychiatry, College of Health Sciences, University of Alberta, Edmonton, Canada; 7 Division of Psychiatry, Haukeland University Hospital, Bergen, Norway; 8 Department of Clinical Medicine, University of Bergen, Bergen, Norway; 9 Department of Biomedicine, University of Bergen, Bergen, Norway

**Keywords:** Genomics, Clinical genetics

## Abstract

Electroconvulsive therapy (ECT) is an effective antidepressant treatment. The mechanisms behind the therapeutic effect are not fully understood, and reliable biomarkers for response are needed. Epigenetic modifications, such as DNA methylation (DNAm), can reflect both genetic and environmental impacts; they may shed light on the mechanisms behind treatment effects and they have the potential to inform response prediction. We performed an epigenome-wide association study (EWAS) in peripheral blood from patients before and after ECT in a Norwegian cohort (n = 65). The methylation levels of 12 differentially methylated CpG positions (DMPs) and 18 differentially methylated regions (DMRs) were significantly associated with percent clinical response. In addition, 29 DMPs and 23 DMRs were significantly associated with remission (Montgomery and Åsberg Depression Rating Scale MADRS < 10 post treatment). Two DMRs were also significantly associated with percent response at baseline and four DMRs were significantly associated with remission at baseline (FDR < 0.05). We did not identify any longitudinal (pre-post) changes in DNAm. We further performed the first meta-analysis (n = 99) between ECT cohorts, combining this Norwegian cohort and a German ECT cohort (n = 34). Seven of the DMRs found to be associated with response in the meta-analyses were previously identified in the Norwegian or the German cohort (FDR < 0.05). Methylation risk scores (MS) calculated using DMPs associated with ECT in the Norwegian cohort showed promising association with response to ECT in the German cohort (p = 0.06). Finally, we found increased neutrophil to lymphocyte ratios, calculated from estimated cell proportions, to be associated with remission (p < 0.003) in the Norwegian cohort.

## Introduction

Electroconvulsive therapy (ECT) is one of the most effective treatments of severe episodes in affective disorders particularly for treatment-resistant depression (TRD; no adequate response to two guideline antidepressant treatments), where approximately 50–70% of patients respond, and 3050% achieve remission [1–3]. However, the underlying mechanisms behind the effects of ECT are not yet fully understood. Most research has focused on neurobiological aspects and a model involving disruption, neuroplasticity, and rewiring of neural circuits [4]. Other mechanisms have been suggested to contribute to the therapeutic effect [5], including altered circulating immune cell proportions [6]. At the molecular level, patients receiving ECT have higher load of genetic risk for major depressive disorder (MDD) [7] compared with patients responding to guideline antidepressant treatments. Epigenetic mechanisms that could reflect the increased genetic and additional risks (e.g. environmental) may play an important role in the putative molecular mechanism underlying ECT effects [8]. Currently, reliable biomarkers to predict ECT response (ECTresp) and elucidate treatment-induced biological changes (ECTexp) are still needed. Clarifying these biological markers may significantly enhance patient selection and treatment efficacy.

DNA methylation (DNAm), a stable and accessible epigenetic modification measurable in peripheral blood, reflects interactions between genetic predispositions and environmental exposures. It has the potential to identify both biomarkers predicting treatment response and molecular changes associated with therapeutic mechanisms [9]. Several studies have identified

DNAm differences associated ECTresp: Studies focusing on candidate genes have found reduced DNAm levels of the *BDNF* promoter [10] and increased methylation levels of the *S100A10* (P11) promoter [11] in blood from patients who respond to ECT, compared to non-responders. Since 2020, two epigenome-wide association studies (EWAS) have reported differentially methylated positions and regions (DMPs, DMRs) associated with ECTresp [12, 13] and ECTexp [12] in relatively small samples (N = 12 [13]; N = 34 [12]). Larger and more comprehensive studies are thus required to confirm these associations, identify reliable biomarkers, and examine whether longitudinal epigenetic changes occur with ECT exposure.

Alterations in circulating immune cell populations have been associated with depression, consistent with dysfunction in both innate and adaptive immunity [14]. The innate and adaptive immune systems influence the risk for depression by interacting with neurotransmitters and neurocircuits [15]. Increased levels of neutrophil counts have been shown to be correlated with the severity of depressive symptoms [16], in line with increased neutrophil to lymphocyte ratio (NLR) in MDD [16–18]. NLR has become an important biomarker for immune system homeostasis, linking the innate and adaptive immune systems [19]. Interestingly, since ECT remitters and non-remitters exhibit differences in their immune profile at baseline, it has been suggested that circulating immune cells could be involved in the acute effect and clinical outcome of ECT[6]. Although DNAm can estimate immune cell proportions, these measures have not yet been evaluated in relation to ECTexp or ECTresp.

To address these gaps, our study investigates epigenetic and immune-system alterations in the largest EWAS on ECT conducted to date, analysing peripheral blood DNAm from a wellcharacterized Norwegian TRD cohort (n = 65) before and after ECT. Specifically, we hypothesize that distinct DNAm patterns will differentiate responders from non-responders (ECTresp), and that ECT exposure itself (ECTexp) will result in measurable epigenetic and immune-cell changes. To enhance statistical power and robustness, we perform a meta-analysis incorporating data from an independent German cohort (total n = 99). Finally, we evaluate the predictive capacity of methylation-based biomarkers, aiming to develop clinically applicable tools to guide personalized treatment decisions and optimize ECT outcomes.

## Materials and methods

### Participants

Patients (n = 65) included in the study were referred to ECT at the Haukeland University Hospital in Bergen, Norway. Patients that were >18 years old and were accepted for treatment were offered to participate. Fifty-two patients had a diagnosis of unipolar depressive disorder (F32.1-F33) and thirteen patients had a diagnosis of bipolar (F31.1-F31.5) depressive disorder, according to the International Classification of Diseases and Related Health Problems 10^th^ revision (ICD-10). Patients unable to give informed consent to the biobank and regional register for neurostimulation treatment were excluded. Patients included gave written consent to the Regional Register for Neurostimulation Treatment in Western Norway, approved by the Norwegian Data Protection Authority (approval no. 2012/5490) and the Regional Biobank for Neurostimulation Treatment (approval no. 2017/925). Combining data from the register and biobank in this observational study, was approved by the Regional Committee for Research Ethics (REK Nord, reference 51089). Most of the patients were on anti-depressant and/or anti-psychotic medication. Seven of 50 patients had a change in antidepressant throughout the ECT treatment, 17 of 51 patients on antipsychotic had a change in antipsychotic medication. The study protocol has been registered at https://clinicaltrials.gov/study/NCT05515159.

### ECT treatment

The protocol used for administering the ECT treatment is described in [20]. The preferred anaesthetics was thiopental (mean dose 3.7 mg/kg, SD 1.0), 3 patients received propofol (mean dose 2.1 mg/kg, SD 1.4). The initial stimulus dose was determined by an age-based method with an average stimulus dose. The stimulus was applied via right unilateral (RUL) electrode placement and with a pulse width of 0.5 ms. Bilateral electrode placement was indicated and used for two patients, and an ultra-brief pulse width (0.25 ms) was indicated and received by one patient. After each treatment, the seizure adequacy was determined based on seizure duration, quality of delta-waves, postictal suppression and postictal reorientation time. If there were signs of an insufficient seizure, adjustments were made, such as increasing the electrical dose or switching from unilateral to bilateral electrode placement. For side effects like cognitive decline, measures included extending treatment intervals, reducing the electrical dose, or using an ultra-brief pulse width. The patients received 3 treatments per week (Monday, Wednesday and Friday), and between 4 and 16 treatments (average 10) in total.

### Clinical assessment

The severity of depressive symptoms was assessed at the start of treatment and within 2–4 days of the final treatment session. The assessments were performed by the treating clinicians using the Montgomery and Åsberg Depression Rating Scale (MADRS) [21] according to routine clinical procedures.

### DNA methylation quantification

Blood samples were taken prior to the first ECT treatment and in conjunction with the final treatment session (in average 8.8 days after last session, standard deviation 7.6). Standard protocols were followed for DNA extraction, and normalization, including measurement of concentration with PicoGreen (Fluorometer), at the Norwegian University of Science and Technology (NTNU) by the Trøndelag Health Study (HUNT) biobank. The DNA samples were run using the Illumina Infinium MethylationEPIC BeadChip v1 (>850 000 CpG sites) at the Life and Brain GmbH, Genomics facility in Bonn.

### Genome-wide quantification of DNAm

The quality control (QC) and preprocessing were performed on the data using the CPACOR pipeline [22], adapted to read Illumina Infinium EPIC files. The steps are briefly summarised as follows. (i) Intensity values were background corrected and values below a detection p-value threshold of 1 × 10^−60^ were set to missing. The detection p-value threshold 1 × 10^−60^ was selected so that <10% of females had a detectable signal on the Y chromosome, as described by [22]. (ii) The proportion of missing values was used to calculate sample and CpG marker call rates. Samples from 2 individuals with a call rate <0.95% were removed. (iii) The intensity values from the six matrices with measured probe type and Cy3/Cy5 colour combinations were quantile-normalised separately. (iv) Beta values from the quantile-normalised intensity values were calculated. (v) Probes with missing values and probes from the X and Y chromosomes were removed. DNAm data from 65 patients before and after the last treatment with ECT remained after QC. Prior to data analyses the beta values were logit-transformed to M values for use in the downstream statistical analysis, as recommended [23].

### Extraction of control probes, estimation of cell proportions and smoking scores

The following were extracted or estimated from the methylation data and included as covariates in the downstream analyses:

(i) Background-corrected intensity values from the Illumina Infinium EPIC control probes were extracted and principal components (PCs) of the control probes were calculated. The first 10 PCs were included in the downstream analyses to account for batch effects.
(ii) The proportions of six different white blood cell types (T lymphocytes (CD4+ and CD8 + ), B-cells (CD19 + ), monocytes (CD14 + ), NK cells (CD56 + ) and neutrophils) were estimated using the Houseman algorithm [24] as implemented in the estimate CellCounts2 function from the R/Bioconductor package *FlowSorted.Blood.EPIC*. This package uses an optimised library of CpGs and reference set described in [25]. Default settings were used. Neutrophils showed the highest variance of inflation (VIF) and were omitted as a covariate from the downstream analyses to avoid issues with collinearity.
(iii) Smoking scores were calculated from the DNAm data, using a method described by [26] and script provided by [27]. Prior to data analyses the beta values were logit-transformed to M values for use in the downstream statistical analysis, as recommended [23].

### Epigenome-wide association study

We applied a mixed linear regression approach, as implemented in the *limma* R package [28], to identify differentially methylated positions (DMPs) associated with

(i) ECTexp (longitudinal change before vs after ECT) (Model 1),
(ii) ECTresp (change in clinical depression score) (Model 1),
(iii) the interaction between ECTexp and ECTresp (Model 2).

Model 1: DNAm ~ ECTexp + ECTresp + covariates

Model 2: DNAm ~ ECTexp + ECTresp + ECTexp:ECTresp + covariates

Patient ID was modelled as a random effect in the mixed linear by specifying it for the block argument of lmFit. The function duplicateCorrelation was used to estimate the correlation between samples from the same individual. Age, sex, estimated smoking scores, estimated blood cell proportions and 10 control probe PCs were included as covariates.

For each analysis, both continuous and binary models were run where ECTresp was coded either as (a) percent response (percent change in MADRS, continuous variable), or (b) remission (MADRS < 10, binary variable).

Additional models were run to examine the relationship between ECTresp and DNAm at baseline: Model 3: DNAm ~ ECTresp + covariates

The significance threshold for DMPs was set to FDR < 0.05.

### Meta-analysis

Meta-analyses using summary statistics from the limma analyses of the Norwegian cohort (described above) and the limma analyses of a German cohort previously described by [12] were performed to identify DMPs supported by both cohorts. The detailed description of the German cohort can be found in [12]. Briefly, 34 patients (age >18) with major unipolar depressive episodes (ICD-10) were recruited for ECT treatment at the Central Institute of Mental Health (CIMH), Mannheim, Germany, between 2014 and 2016. ECT was prescribed for treatment-resistant depression, prior positive ECT response, or severe depression with psychotic features, suicidality, or refusal of food/fluid. Patients received s-ketamine (~1.0 mg/kg) and succinylcholine ( ~ 1.0 mg/kg) for anesthesia and muscle relaxation. Seizure thresholds were determined during the first session, with subsequent treatments delivered at >2.5 times the threshold. Stimuli were increased if seizures were insufficient or clinical response was lacking. Patients underwent 2–3 sessions per week, beginning with unilateral stimulation, with optional switch to bilateral based on clinical judgment. Exclusion criteria included substance use disorders (excluding tobacco and alcohol) and lifetime schizophrenia. All patients were of European ancestry and maintained stable medication during treatment. Blood was collected before and after treatment similar to the timepoints in the Norwegian cohort. DNAm was obtained using the same array in the same facilities and was QC with the same protocol as the Norwegian cohort.

Since clinical assessment was done with HAMD scale in the German cohort and MADRS in the Norwegian cohort, we tested the correlation by converting MADRS scores following established protocol [29, 30]. We confirmed the high correlation between the two scores both in correlation value and in the classification of responders / non responders (see supplementary Figure 1) and therefore proceeded with the meta-analyses.

In the German study ECTresp was defined as (i) delta score (change in clinical depression score) and (ii) binary response, where response was defined as at least 50% reduction in the depression score. Therefore, additional EWASs were performed in the Norwegian cohort using the same ECTresp definitions as in the German study: DMADRS for the continuous model, and >= 50% reduction in MADRS for binary model. The correlation between %MADRS and DMADRS was 0.9.

For the binary analysis 19 patients with 50% reduction had MADRS > = 10 and were thus not in remitters category as analysed in the Norwegian cohort only.

Metagen from the R package *meta* [31] was used to perform the meta-analyses. Effect sizes and standard errors from each cohort were used to test for significance using a fixed effects model.

### Differentially methylated regions

Differentially methylated region (DMR) analyses were performed using comb-p [32] on the pvalues from the EWAS and meta-analyses, specifying parameters as seed p-value = 0.001 and maximum distance between probes of 750 base pairs, as recommended [33]. DMRs were analysed using the p-values obtained from the EWAS on ECTresp, ECTexp, interaction between ECTresp*ECTexp and ECTresp at baseline. Since ECTresp was coded as (a) percent response and (b) remission in the EWAS on the Norwegian cohort, and (i) delta score and (ii) binary response in the meta-analyses, DMR analyses were run for each of them. A Šidák correction was applied to adjust for multiple testing, and adjusted p-value of 0.05 level was used as the significance threshold.

DMPs and DMRs were annotated using the Bioconductor package *IlluminaHumanMethylationEPICanno.ilm10b4.hg19*.

### Analyses of estimated cell-type proportions

Following the same rationale as for the EWAS, a mixed linear regression approach was used to test NLR and the estimated cell-type proportions (neutrophils, B-cells, monocytes, CD4T, CD8T and natural killer cells (NK)) for association with (i) ECTexp (longitudinal change before vs after ECT), (ii) ECTresp (change in clinical depression score) and (iii) the interaction between ECTresp and ECTexp. As for the EWAS, separate models were run where response was coded as (a) percent response and (b) remission. Patient ID was modelled as a random effect in the mixed linear model and, age, sex, estimated smoking scores, and 10 control probe PCs were included as covariates.

### Calculation of methylation scores (MS)

In the same way that polygenic risk scores summarise the joint effect of multiple genetic variants, MS can be calculated to include the effect of multiple CpGs. We calculated a MS for response to ECT (MS-ECT) derived from the bigger Norwegian cohort to test if it was associated with ECTresp in the German cohort. The DMPs found to be associated with ECTresp in the Norwegian cohort were used to calculate MS-ECT for the individuals in the German cohort. The score was calculated as a weighted sum of beta values from the German cohort, weighted by the effect sizes from the EWAS on the Norwegian cohort. To handle possible correlations between the DMPs, a pipeline made available by [34] was used for the calculation of methylation scores. Briefly, this pipeline uses *CoMeBack* [35] to define co-methylated regions with a correlation > 0.3 and chooses the DMP with the most significant p-value to represent the region. The DMPs representing the co-methylated regions are then combined with the DMPs not in such regions (singletons). Next, multiple MSs were calculated across different p-value thresholds: 5 × 10^−2^, 5 × 10^−3^, 5 × 10^−4^ and 5 × 10^−5^. Finally, regression analysis was used to test whether the calculated MS was associated with the delta score and binary response.

In addition, an MS for MDD (MS-MDD) was calculated for the individuals in the Norwegian cohort to test if it was associated with ECTresp. The MS-MDD was derived from the weighted sum of the 15 DMPs that showed association with MDD (p < 6.43 × 10^−8^) in [36]. CoMeBack considers correlation between EPIC array probes that are <2 kb apart if there is a chain of genomic CpGs between them which are at most 400 bp apart. The shortest distance between any of the 15 DMPs associated with MDD that were on the same chromosome was 13 Mb. The weighted sum was therefore calculated directly without consideration of correlation. The beta-values from the Norwegian cohort were residualised for age, sex, smoking score and 10 control probe PCs prior to the calculation of MS-MDD, and the effect sizes from the study in [36] were used as weights.

Linear regression was used to test the calculated MS-MDD for association with percent response and remission.

### Pathway analyses

Pathway analysis was performed using the R/Bioconductor package *missMethyl* [37]. DMRs with a Šidák corrected p-value < 0.05 and at least 4 CpGs long were submitted for pathway analysis using the function GOregion. Pathway or gene set size has been shown to affect the statistics of overrepresentation analyses [38]. Small gene sets may therefore be sensitive to inflated enrichment, while large gene sets may be unspecific and difficult to interpret. We therefore removed gene sets with less than 15 and more than 500 genes. The remaining gene sets with a p-value < 0.01 were clustered based on overlapping significant genes. Jaccard distance was calculated between all pairs of the gene sets and the gene sets were clustered using complete linkage agglomerative hierarchical clustering. Finally, heatmaps were created using the effect sizes of the DMPs with the lowest pvalue mapping to the significant genes in the gene set.

All scripts can be made available upon request.

## Results

### Clinical assessment of ECT treatment in the Norwegian cohort

Cohort demographic and clinical responses are summarised in Table 1. There was no difference between responders or remitters and non-responders for medication use or for number of ECT sessions, thus those were not corrected for. The average age of patients was 50, and 43% of the patients were male (see Table 1). There was a significant difference between responders and non-responders for age, which was corrected for. Comorbidities were not systematically recorded and were thus not corrected for. Although sex was not different between the groups, it was still corrected because it has a major effect on DNAm.

**Table 1:** Sample overview.

	Total	Non-responder	Responder	Remitter	p^a^
n	65	21	44	25	
Sex = M (%)	28 (43.1)	7 (33.3)	21 (47.7)	10 (40.0)	0.408
Age (mean (SD))	50 (16.8)	42.81 (16.01)	53.43 (16.20)	52.56 (17.44)	0.016
MADRS_before (mean (SD))	35.09 (6.7)	33.95 (5.38)	35.64 (7.25)	34.76 (7.47)	0.348
MADRS_after (mean (SD))	14.02 (8.6)	24.19 (5.44)	9.16 (4.69)	5.92 (2.69)	<0.001
MADRS_percent (mean (SD))	59.33 (24.8)	28.65 (12.58)	73.98 (12.76)	82.43 (8.54)	<0.001
MADRS_delta (mean (SD))	21.08 (10.2)	9.76 (4.62)	26.48 (7.31)	28.84 (7.30)	<0.001
No of ECT treatments (mean (SD))	10.23 (3.7)	11.43 (3.91)	9.35 (2.88)	8.15 (2.58)	0.126
Anti-depressant n (%)	50 (76.9)	15 (71.4)	35 (79.5)	20 (80.0)	0.681
Antipsychotic n (%)	51 (78.5)	17 (81.0)	34 (77.3)	19 (76.0)	0.988
Anticonvulsant n (%)	3 (4.6)	0 (0.0)	3 (6.8)	1 (4.0)	0.553
Lithium n (%)	7 (10.8)	3 (14.3)	4 (9.1)	2 (8.0)	0.838

^a^The p-value refers to the comparison between the responders and non-responders.

**Fig. 1:**
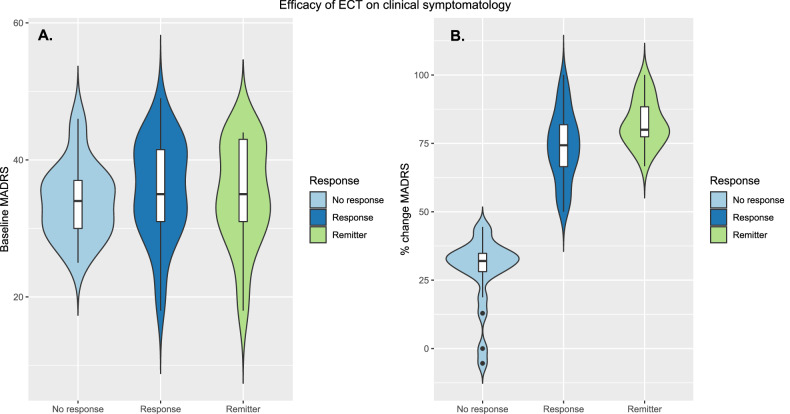
Efficacy of ECT on depression symptoms. **A** MADRS score at baseline for responders, non-responders and remitters. **B** Distribution of percent change in MADRS for responders, non-responders and remitters.

Mean depression score (MADRS) before treatment was 35.1 and ranged between 18 and 49. Sixtythree (96.9%) patients showed reduced MADRS scores after treatment. Forty-four (67.7%) patients were classified as responders and 25 (38.5%) patients were classified as remitters. MADRS at baseline was similar in non-responders, responders, and remitters (Fig. 1A). The median reduction in MADRS was 32.1% and 74.4% for non-responders and responders, respectively, while the median reduction for subgroup of responders that reached remission was 80.0% (Fig. 1B).

### DNAm patterns associated with treatment and response to ECT

We used longitudinal linear mixed effects models that account for both baseline and post-treatment DNAm, using percent response or remission as a measure of the response to treatment.

#### Differentially methylated position analysis

The top 10 DMPs are presented in Table 2. All DMPs and additional details about the DMPs are presented in Supplementary Tables (see respective paragraphs).

**Table 2:** Top 10 DMPs associated with ECTresp (percent response and remission) in the Norwegian cohort.

CpG	gene	logFC	P-value	FDR
**Percent response across timepoints**				
cg25117092	*MED12L;P2RY14*	−0.011	4.82E-08	0.022
cg13221347	*STK32C*	−0.011	8.19E-08	0.022
cg25883333	*MEGF11*	−0.009	1.03E-07	0.022
cg15821939	*MIR596*	0.012	1.46E-07	0.022
cg25673948	*MIR596*	0.013	1.62E-07	0.022
cg09177567	*MIR596*	0.028	2.28E-07	0.025
cg12735254	*STK32C*	−0.008	4.12E-07	0.034
cg05650680	*MIR596*	0.007	4.53E-07	0.034
cg02099337		0.015	4.82E-08	0.034
cg19560656		0.008	8.19E-08	0.039
**Remission across timepoints**				
cg01722829		−0.315	2.32E-09	0.001
cg07160746	*KCNS1*	−0.420	5.45E-09	0.001
cg18677860		−0.374	6.14E-09	0.001
cg06183427	*IRX4*	−0.414	1.47E-08	0.002
cg02956519		0.523	1.70E-08	0.002
cg24078577	*ASRGL1*	−1.186	3.93E-08	0.004
cg17293936	*DGKG*	−0.508	9.16E-08	0.008
cg04391789	*ZNF423*	0.327	1.01E-07	0.008
cg14157435	*NRP2*	0.818	1.64E-07	0.012
cg09929786		0.407	2.03E-07	0.013

Association with percent response: 12 DMPs had an FDR value < 0.05, 4 of which are annotated to *MIR596* (MicroRNA 596) (Supplementary Table 1).

Association with remission: 29 DMPs had an FDR value < 0.05 (Supplementary Table 2).

No effects of ECTexp on DNAm were found between the two time points, in either the percent response or the remission model (Supplementary Table 3 and 4). No significant interaction between ECTexp and ECTresp were found in either the percent response or the remission model (Supplementary Table 5 and 6).

#### Differentially methylated region analysis

**Fig. 2:**
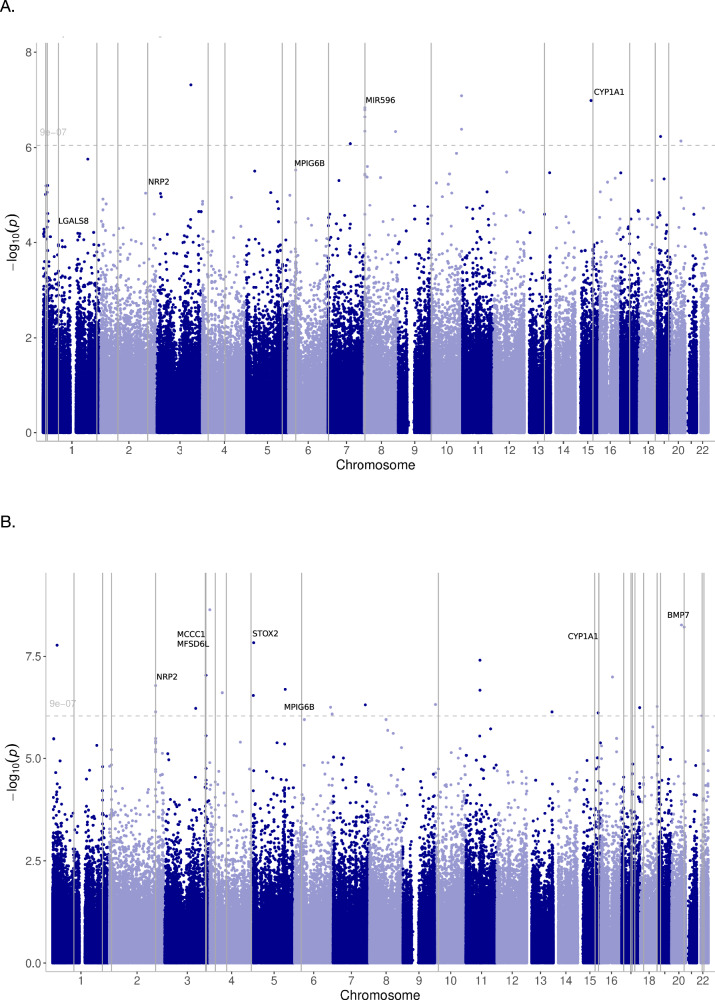
Manhattan plots showing the significant DMRs. DMRs with 8 or more CpGs are annotated with gene names. The horizontal dotted line indicates the epigenome-wide significance threshold (p = 9 × 10^−7^). Responses are coded as (**A**) percent change in MADRS and (**B**) remission.

**Fig. 3:**
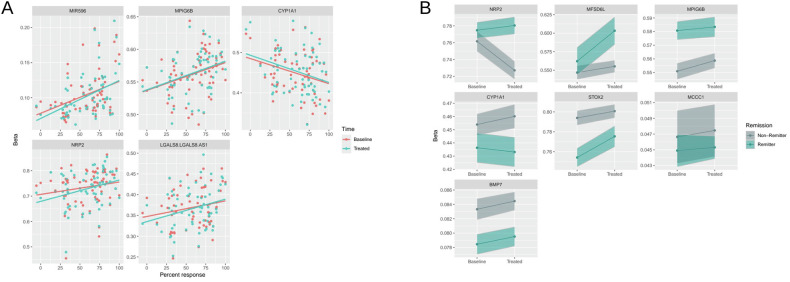
DNAm vs ECTresp of the longest DMRs. **A** DNAm vs percent response. Separate regression lines represent the association before and after treatment and show similar association with response at both time points. There is no significant difference between the regression lines before (baseline) and after treatment. **B** DNAm levels in remitters and non-remitters. The lines represent the average difference in methylation level between the two time points and the bands represent the standard error of the mean. The plots show a clear difference between remitters and non-remitters. Some of the lines suggest a small difference in DNAm levels before (baseline) vs after treatment, but these differences are not significant. The plots were made on data that were cleaned with the function limma::removeBatchEffects to remove the effect of all covariates that were included in the EWAS.

The top 10 DMRs are presented in Table 3. All DMRs and additional details about the DMRs are presented in Supplementary Tables (see respective paragraphs). Figure 2A and B display Manhattan plots of DMRs for ECTresp and remission, respectively, while Fig. 3 illustrates the longitudinal changes in average DNAm associated in the longitudinal model of percent response (Fig. 3A) and remission (Fig. 3B).

**Table 3:** Top 10 DMRs associated with ECTresp (percent response or remission) in the Norwegian cohort, across timepoints or at baseline.

chromosome	start	end	min_p	n_probes	z_p	z_sidak_p	genes
**Percent response across timepoints**							
8	1765217	1765820	5.491E-19	8	1.465E-27	1.616E-24	*MIR596*
6	31691198	31692260	1.608E-16	24	2.11E-24	1.322E-21	*MPIG6B*
15	75019070	75019376	1.464E-11	10	7.77E-16	1.69E-12	*CYP1A1*
2	206628415	206628773	1.047E-08	9	1.201E-11	2.232E-08	*NRP2*
1	67600547	67600835	9.595E-08	5	1.03E-11	2.38E-08	*C1orf141*
1	236686564	236686988	9.601E-07	10	1.266E-10	1.986E-07	*LGALS8; LGALS8-AS1*
17	40274524	40274811	6.815E-07	7	9.269E-11	2.149E-07	*HSPB9; KAT2A*
10	695844	696063	5.046E-06	5	4.778E-10	1.451E-06	*DIP2C; C10orf108*
4	25090491	25090665	1.15E-05	4	1.228E-09	4.694E-06	
4	99851003	99851281	1.617E-05	7	2.652E-09	6.348E-06	*EIF4E*
**Remission across timepoints**							
2	206628355	206628773	5.406E-25	10	7.416E-29	1.18E-25	*NRP2*
3	185911316	185912253	3.338E-12	6	2.579E-21	1.831E-18	*DGKG*
17	8702369	8702896	9.381E-14	8	8.307E-18	1.049E-14	*MFSD6L*
18	74799250	74799572	6.076E-12	4	1.808E-16	4.587E-13	*MBP*
6	31691354	31692026	1.962E-09	18	4.428E-15	4.397E-12	*MPIG6B*
10	14372108	14372913	3.449E-05	5	1.053E-13	8.699E-11	*FRMD4A*
1	223566447	223566794	5.052E-10	7	3.332E-12	6.388E-09	*C1orf65; CCDC185*
2	9613721	9614052	3.366E-08	5	3.18E-12	6.392E-09	*IAH1*
5	476405	476646	1.922E-08	3	1.729E-11	4.773E-08	*SLC9A3; LOC100288152*
18	14748040	14748298	3.606E-08	7	6.964E-11	1.796E-07	*ANKRD30B*
**Percent response at baseline**							
6	31691198	31691812	3.677E-07	17	5.242E-12	5.68E-09	*MPIG6B*
8	1765217	1765477	3.35E-07	6	2.96E-12	7.574E-09	*MIR596*
**Remission at baseline**							
18	74799250	74799572	1.573E-10	4	6.413E-16	1.376E-12	*MFSD6L*
5	178986372	178986830	3.391E-08	11	4.682E-13	6.801E-10	*RUFY1*
17	8702698	8702896	1.597E-07	5	1.784E-09	5.994E-06	*MFSD6L*
6	31691506	31691870	0.0001203	9	2.652E-07	0.0004846	*MPIG6B*

DMRs associated with percent response: 18 DMRs with 4 or more CpGs and an adjusted p-value < 0.05 were found using the p-values for percent response extracted from the longitudinal linear mixed model (Supplementary Table 7). The longest DMRs (8 or more CpGs) were annotated to the *MPIG6B* (Megakaryocyte and Platelet Inhibitory Receptor G6b), *CYP1A1* (Cytochrome P450 Family 1 Subfamily A Member 1), *LGALS8;LGALS8-AS1* (Galectin 8;LGALS8 Antisense RNA 1), *NRP2* (Neuropilin 2) and *MIR596* genes.

DMRs associated with remission: 23 DMRs with 4 or more CpGs and an adjusted p-value < 0.05 were found using the p-values for remission extracted from the longitudinal linear mixed model (Supplementary Table 8). The longest DMRs (8 or more CpGs) associated with remission are annotated to *MPIG6B*, *CYP1A1*, *NRP2, BMP7* (Bone Morphogenetic Protein 7)*, MCCC1* (Methylcrotonyl-CoA Carboxylase Subunit 1)*, STOX2* (Storkhead Box 2) and *MFSD6L* (Major Facilitator Superfamily Domain Containing 6 Like).

The DMRs associated with percent response and remission show similar DNAm levels across both time points (Fig. 3).

No DMRs were found to be associated with the effect of ECTexp.

### DNAm patterns associated with response at baseline

We also analysed the association between DNAm at baseline (i.e. accounting for different levels in DNAm at baseline only) and ECTresp. No DMPs were found to be significantly associated with percent response or remission using baseline DNAm (Supplementary Tables 9 and 10). Two and four DMRs were significantly associated with percent response and remission, respectively (Table 3). The two DMRs associated with percent response (*MIR596* and *MPIG6B*) and three of the DMRs associated with remission (*MPIG6B* and 2 x *MFSD6L*) were also identified in the DMR analyses in the longitudinal linear mixed models (i.e. with both timepoints). In addition, one DMR annotated to *RUFY1* (RUN And FYVE Domain Containing 1) was found to be associated with remission at baseline.

### Analysis of alterations in estimated immune system blood cell composition

Immune cell type proportions as well as NLR were estimated from the DNAm and analysed for their association with either the percent response or with remission (Table 4 and Supplementary Figure 3).

**Table 4:** Association of immune cell type proportions with ECTexp and ECTresp (percent or remission).

	Percent response			ECTexp			Percent response x ECTexp interaction		
Cell type	Estimate	SE	P-value	Estimate	SE	P-value	Estimate	SE	P-value
NLR	5.57E-03	2.78E-03	5.03E-02	1.04E-01	8.05E-02	2.01E-01	-3.30E-03	3.07E-03	2.86E-01
CD4T	−1.11E-04	2.30E-04	6.30E-01	−7.78E-03	6.54E-03	2.38E-01	2.64E-04	2.49E-04	2.92E-01
CD8T	−4.09E-04	1.91E-04	**3.68E-02**	−4.80E-03	3.39E-03	1.61E-01	2.31E-05	1.20E-04	8.48E-01
Neu	8.66E-04	4.31E-04	**4.96E-02**	1.82E-02	1.25E-02	1.48E-01	-5.15E-04	4.74E-04	2.82E-01
Mono	−9.01E-05	1.24E-04	4.71E-01	−6.33E-04	2.74E-03	8.18E-01	1.08E-04	1.00E-04	2.87E-01
Bcell	−1.22E-04	8.27E-05	1.44E-01	−3.58E-03	1.78E-03	**4.69E-02**	8.52E-05	6.44E-05	1.91E-01
NK	−1.26E-04	1.05E-04	2.39E-01	−7.28E-04	3.30E-03	8.26E-01	7.38E-05	1.28E-04	5.65E-01
	**Remission**			**ECTexp**			**Remission x ECTexp interaction**		
**Cell type**	**Estimate**	**SE**	**P-value**	**Estimate**	**SE**	**P-value**	**Estimate**	**SE**	**P-value**
NLR	3.86E-01	1.26E-01	**3.36E-03**	1.03E-01	8.04E-02	2.04E-01	−2.26E-01	1.55E-01	1.48E-01
CD4T	−1.08E-02	1.08E-02	3.21E-01	−7.79E-03	6.54E-03	2.37E-01	1.68E-02	1.25E-02	1.86E-01
CD8T	−1.81E-02	9.19E-03	5.34E-02	−4.79E-03	3.39E-03	1.61E-01	3.44E-03	6.08E-03	5.74E-01
Neu	6.31E-02	1.93E-02	**1.89E-03**	1.80E-02	1.24E-02	1.51E-01	−3.36E-02	2.40E-02	1.66E-01
Mono	−1.49E-02	5.61E-03	**1.02E-02**	−7.13E-04	2.71E-03	7.93E-01	8.59E-03	5.01E-03	9.15E-02
Bcell	−7.87E-03	3.86E-03	**4.62E-02**	−3.60E-03	1.77E-03	**4.56E-02**	3.48E-03	3.29E-03	2.95E-01
NK	−1.17E-02	4.80E-03	**1.81E-02**	−7.22E-04	3.30E-03	8.27E-01	3.82E-03	6.48E-03	5.57E-01

^*^NLR neutrophil-to-lymphocyte ratio, *CD4T* CD4 + T-cells, *CD8* CD8 + T-cells, *Neu* neutrophils, *Mono* monocytes, *Bcell* B-cells, *NK* natural killer cells.

Significant associations are highlighted in bold.

In the analysis of the association with percent response: we found a higher proportion of CD8T cells (p-value = 0.0368) and a lower proportion of neutrophils (p-value = 0.0496) with ECTresp. We found a decrease in B-cells associated with ECTexp (p-value = 0.0469).

In the analysis of the association with remission: We found a higher proportion of neutrophils (p-value = 0.0019) and a lower proportion of monocytes (p-value = 0.010), B-cells (p-value = 0.046) and NK cells (p-value = 0.018) to be associated with remission. While a decrease in B-cells (p-value = 0.0456) was associated with ECTexp.

There were no associations in the interaction models of ECTexp*ECTresp for percent response nor remission.

### Meta-analyses between the Norwegian and German cohorts

We performed a meta-analysis between the previously described German cohort (N = 34 [12]) and the Norwegian cohort described above (N = 65). Patients in both cohorts were exposed to similar treatment. In the German cohort response to treatment was measured using ΔHAMD, while in the Norwegian cohort response to treatment was measured using per ΔMADRS. Before meta-analysis, we confirmed that MADRS scores were comparable to HAMD21 (Supplementary Figure 1) and proceeded with the meta-analysis using ΔMADRS in the Norwegian cohort and ΔHAMD in the German cohort for the continuous model. For the binary model, due to the smaller German cohort, the category was defined based on reduction in depression symptoms above or below 50%.

In the delta score response model, we found association with 19 DMPs (FDR < 0.05) and 15 DMRs (adjusted p-value < 0.05; Tables 5 and 6, and Supplementary Tables 11 and 12). Five of the 15 DMRs were previously identified in the Norwegian (percent change in MADRS) or German (ΔHAMD) cohorts. No significant effects were found for ECTexp (longitudinal effects) or the interactions between ECTexp and ECTresp (Supplementary Table 13 and 14 respectively).

**Table 5:** DMPs associated with ECTresp (delta score or binary response) in the meta-analysis.

CpG	gene	logFC	P-value	FDR
**Delta score across timepoints**				
cg01444849		−0.018	4.68E-09	0.003
cg02099337		0.036	2.86E-08	0.009
cg05504927	*RUNX1*	0.020	6.15E-08	0.012
cg12726743	*CAMK2G*	0.009	9.04E-08	0.012
cg25117092	*MED12L; P2RY14*	−0.023	9.86E-08	0.012
cg12239753	*SAA2-SAA4*	−0.013	2.72E-07	0.025
cg14223001		0.028	2.96E-07	0.025
cg25668052	*GLG1*	−0.018	3.70E-07	0.027
cg11077186	*LINC01141*	0.020	3.97E-07	0.027
cg24854181		0.023	4.68E-09	0.031
**Binary response across timepoints**				
cg03803796		0.336	2.08E-08	0.013
cg09177567	*MIR596*	1.364	5.34E-08	0.016
cg06661232	*CACNG3*	0.399	1.11E-07	0.020
cg14223001		0.597	1.31E-07	0.020
cg08179817		−0.327	2.67E-07	0.032
cg20249210		0.346	3.47E-07	0.035
cg24709471	*CRABP1*	−0.515	4.34E-07	0.036
cg12735254	*STK32C*	−0.378	4.87E-07	0.036
cg12746557	*KCNJ15*	0.653	5.35E-07	0.036
cg25117092	MED12L; P2RY14	−0.464	6.16E-07	0.037
**Delta score baseline**				
cg16155903		−0.0195	9.92E-09	0.0060
cg13839160	*GNG7*	−0.0214	1.07E-07	0.0283
cg15407190		−0.0192	1.41E-07	0.0283
**Binary score baseline**				
cg20063965	*BAI1*	−0.545	1.51E-08	0.0091
cg07751528	*ROR2*	−0.333	2.09E-07	0.0372
cg03803796		0.359	3.09E-07	0.0372
cg25376962	*GRIK4*	−1.079	3.13E-07	0.0372
cg09904793	*PRDM16*	0.412	3.68E-07	0.0372
cg12833948	*BAIAP3*	−0.458	3.70E-07	0.0372

The Top 10 are displayed for analyses across timepoints, the significant DMPs are displayed for baseline.

**Table 6:** DMRs associated with ECTresp (delta score or binary response) in the meta-analysis.

chromosome	start	end	min_p	n_probes	z_p	z_sidak_p	genes
**Delta score across timepoints**							
15	75019070	75019376	8.30E-12	10	1.25E-16	2.19E-13	*CYP1A1*
8	1765217	1765477	5.97E-11	5	1.48E-15	3.35E-12	*MIR596*
10	695844	696063	3.89E-09	5	1.16E-13	3.20E-10	*DIP2C;C10orf108*
1	67600547	67600963	1.35E-07	6	8.09E-12	1.17E-08	*C1orf141*
21	42741788	42742432	0.0001837	5	2.77E-11	2.59E-08	*MX2*
13	112986154	112986978	0.005391	4	8.75E-11	6.40E-08	
11	44978644	44979029	5.98E-07	4	7.06E-11	1.11E-07	
8	1321333	1321883	0.00175	5	4.83E-10	5.30E-07	
6	291687	292596	0.01001	7	1.09E-09	7.23E-07	*DUSP22*
21	39644216	39644354	3.93E-06	6	2.28E-10	9.96E-07	*KCNJ15*
**Binary response across timepoints**							
6	291687	292596	6.33E-07	8	3.44E-19	2.28E-16	*DUSP22*
8	1765217	1765477	8.23E-13	5	6.47E-18	1.50E-14	*MIR596*
19	50666238	50666552	5.67E-11	5	1.47E-15	2.77E-12	*C19orf41*
6	31691198	31692026	1.09E-06	18	7.54E-12	5.49E-09	*C6orf25*
2	43295303	43295595	2.55E-07	4	1.02E-11	2.10E-08	
6	168785320	168785524	4.57E-07	4	9.86E-12	2.92E-08	
11	44978644	44979029	5.66E-07	4	4.03E-11	6.31E-08	
1	67600547	67600835	6.98E-07	5	4.20E-11	8.79E-08	*C1orf141*
12	2944163	2944493	5.21E-06	8	3.83E-10	6.99E-07	*NRIP2*
7	123198201	123198553	1.02E-05	6	8.50E-10	1.46E-06	*NDUFA5*
**Delta score baseline**							
2	3699195	3699353	3.93E-07	4	6.27E-12	2.39E-08	
**Binary response baseline**							
2	3699195	3699563	1.24E-08	5	7.74E-14	1.27E-10	
6	291687	292522	0.0002098	6	3.23E-13	2.33E-10	*DUSP22*
8	144660772	144661051	3.07E-07	5	4.22E-12	9.13E-09	*NAPRT1;NAPRT*
22	38092771	38092989	6.48E-05	5	5.71E-07	0.001579	*TRIOBP*

The Top 10 are displayed for analyses across timepoints, the significant DMPs are displayed for baseline.

In the binary model, 14 DMPs had an FDR < 0.05 and 20 DMRs an adjusted p-value < 0.05 (Tables 5 and 6, and Supplementary Tables 15 and 16). Six of the 20 DMRs identified were associated in the Norwegian cohort analyses. No significant effects from ECTexp alone were found on DNAm, while one CpG showed significant interaction between ECTexp and ECTresp (Supplementary Table 17 and 18).

Meta-analysis of DNAm at baseline: 3 DMPs were significantly associated with delta score response and 6 DMPs associated with binary response (Table 5, Supplementary Table 19 and 20 respectively). One intergenic DMR was associated with both delta score and binary response and three additional DMRs were associated with binary response (Table 6, Supplementary Table 21).

### Association between methylation scores and response to treatment with ECT

We tested if a methylation score (MS) derived from ECTresp (continuous and binary models) in the Norwegian cohort could be associated with ECTresp in the German cohort. We derived a MSECTresp using weights from DMPs with a p-value < 1 × 10^−4^ in the Norwegian cohort at baseline.

The MS-ECTresp showed a trend towards association with ECTresp (p < 0.06) in the German cohort for the continuous model (Supplementary Table 22).

We also tested whether MS derived from DNAm associated with MDD was associated with response to ECT. We derived a methylation score from a recently published EWAS for MDD [36]. There were no significant differences in MS for MDD, associated with percent response or remission after ECT.

### Pathway analysis

We tested the genes associated with the identified DMRs for enrichment with gene ontology (GO) terms [39]. The DMRs associated with ECTresp (percent response) were enriched for 22 GO terms (nominal p-value < 0.01). The gene ontology terms showed a high level of overlap in their member genes and could be grouped into 4 clusters involving: “modulation of chemical synaptic transmission”, “carbohydrate derivative catabolic process”, “regulation of synapse organisation” and “forebrain development” (Supplementary Figure 3).

The DMRs associated with remission in the Norwegian cohort, and the delta depression score and binary response from the meta-analyses were enriched in 31, 4 and 21 gene ontology terms, respectively (nominal p-value < 0.01). Each list of gene ontology terms could be clustered into 4, 1 and 3 groups of ontology terms based on their member genes (Supplementary Figures 4–6).

## Discussion

In this study we have investigated epigenome-wide DNAm levels associated with exposure to ECT (ECTexp) and response to ECT (ECTresp). We identified several DMPs and DMRs associated with ECTresp. We also found significant reduction in estimated B-cell proportions post treatment and significant associations between ECTresp and various cell type proportions. Remarkably, we did not identify changes associated with ECTexp which might reflect that effects associated with response can be captured in peripheral tissues, but effects of the exposure might be brain specific.

We used two models for ECTresp: a continuous model based on change in depression scale, which is preferred for statistical power, and a binary model where patients are categorised, which is more relevant for clinicians. We compared remitters to non-remitters in the bigger cohort as this is the most clinically meaningful category, while in the meta-analysis due to the small cohort size of the German cohort we used a cut-off of 50% reduction in depression to define the categories.

Significant DMPs and DMR associated across different ECTresp in the Norwegian cohort and the meta-analysis, were annotated to the gene *MIR596*, a short noncoding RNA. The DMPs annotated to *MIR596* are part of the DMR which spans the entire gene. Increased methylation levels of the promoter could indicate lower expression of *MIR596*, which in turn could lead to down-regulation of gene expression. In the literature, epigenetic inactivation of *MIR596* is associated with prostatic cancer, while its overexpression has been shown to deregulate Wnt-β-catenin signaling [40]. Increased Wnt-β-catenin signaling has been observed after repeated electroconvulsive seizures in rat hippocampus, which could contribute to the therapeutic action [41]. We further explored the genomic region of this DMR in the ENCODE dataset, using the ENCODE screen genome browser (https://screen.wenglab.org [42, 43]). This region overlaps with a promoter (EH38E3814373) which encompasses a single nucleotide variant (rs61388742) associated with expression of the gene *CLN8* (Ceroid Lipofuscinosis 8). Mutations in *CLN8* have been implicated in a severe form of childhood generalized tonico clonic epilepsies (OMIM: 607837). In a mouse model of kindling epilepsy (electrical shock induced epilepsy), the expression of *cln8* was up-regulated in hippocampus [44]. The up-regulation of *CLN8* in response to electrical stimulation could indicate its involvement in the brain’s adaptive mechanisms to such stimuli or the seizure itself. Visual inspection of the DNAm levels associated with percent response indicates that there is a sub-group of participants with at least 25% response that have a higher methylation level of this DMR (Fig. 3). Given the potential relevance of the regulation of this gene for ECTresp, further investigations will be required to determine if genetic variations in the DMR could regulate the methylation and thus the expression of linked genes and their relevance in response to ECT treatment. Interestingly, in a recent study Kaurani et al. [45] characterised miRNA associated with ECTresp in their sample (N = 64). *MIR596* was not among the miRNA associated with ECTresp, which might be a type I/II error in one of the studies, but might also support that the DMPs/DMRs identified in our study regulates the expression of *CLN8* rather than *MIR596*, this will warrant further investigation.

Two DMPs associated with ECTresp were annotated to *STK32C*, a protein kinase which was shown to display differences in methylation in monozygotic twins discordant for depression [46].

The most significant DMR associated with remission is annotated to *NRP2. NRP2* encodes a transmembrane receptor protein that interacts with Vascular Endothelial Growth Factor (VEGF), a growth factor implicated in the response to antidepressant treatment and in response to ECT [47]. The DMR overlaps exon 13 of *NRP2*. Changes in DNAm of exons has been shown to affect alternative splicing [48]. Our results may therefore indicate that alternative usage of exon 13 affects how NRP2 interacts with VEGF and thereby response to treatment with ECT.

In this study, the DMR spanning the highest number of probes overlaps the first 3 exons of *MPIG6B* and shows increased DNAm with increased ECTresp (Table 3 and Supplementary Figure 2). *MPIG6B* is a critical regulator of megakaryocyte maturation [49] and platelet biogenesis [50]. It is an inhibitory receptor, which inhibits platelet aggregation and activation. Platelets have been shown to have altered function in several mental disorders [51–53] and are considered to link mental disorders to immunological and coagulation-related disorders, including cardiovascular disorder [54]. Pathway analysis of the DMRs in remitters (Supplementary Figure 5) highlighted two other genes with reduced methylation, *DGKG* (Diacylglycerol Kinase Gamma), involved with platelet activation, and *PF4* (Platelet Factor 4), involved in megakaryocyte differentiation and in cognition [54]. *MPIG6B* has also been suggested to be involved in regulation of immune response by CD4 + T cells [55].

The meta-analysis identified several DMPs and DMRs that were previously identified in the Norwegian and German cohorts. These included the *CYP1A1*, *MIR596*, *MPIG6B* and *BLCAP*;*NNAT*, supporting their association with ECTresp. In addition, the meta-analyses identified several DMRs that were not picked up by the single cohorts. Of these, the most significant DMR associated with binary response was found to overlap the promoter and first exon of *DUSP22* (Dual Specificity Phosphatase 22) (Supplementary Table 16), and the most significant DMR associated with delta score response was found to overlap the transcriptional start site (TSS) of *KCNJ15 (*Potassium Inwardly Rectifying Channel Subfamily J Member 15). Dual-specificity phosphatases (DUSPs) have been shown to modulate diverse neuronal functions and have been associated with mental and neurological disorders [56, 57]. *KCNJ15* was recently shown to be downregulated in patients with epilepsy and shown to be co-expressed with other genes associated with epilepsy in human brain tissue [58]. Common and rare variants in *KCNJ15* and other K-channels are associated with several neuropsychiatric disorders and are important treatment targets [59].

DMRs annotated to *MPIG6B* and *MIR596* were also found to be associated with ECTresp in baseline DNAm samples. The identification of DMRs associated with ECTresp at baseline is promising with regards to being able to predict response before the initiation of treatment. This was further underlined by the calculation of MS-ECT, which also approached significant association with the delta score response at baseline.

Four different response measures were used in this study (percent response and remission for the Norwegian cohort and delta score and binary response for the meta-analysis). Although there was an overlap in the DMRs identified using the four different response measures, none of the DMRs were found by all measures. This suggests that the models identify different signals associated with ECTresp. The continuous response measures (percent response and delta score) identify associations where the degree of change in methylation is correlated with the degree of clinical response and will therefore point to molecular changes that are important for response. The binary response measures (binary response and remission) identify methylation changes associated with larger clinical responses and may therefore be more clinically relevant.

Alterations in circulating immune cells have been observed in depression [14], and our results support the hypothesis that such immune changes may also be relevant to the effects of ECT. We found a reduction in B-cell proportions associated with ECT exposure and increased neutrophil-tolymphocyte ratio (NLR) and neutrophil proportions associated with remission. These findings suggest that both innate and adaptive immune responses may be modulated during ECT and could potentially contribute to treatment efficacy. Importantly, changes in immune parameters have previously been reported following ECT, including transient increases in pro-inflammatory cytokines after a single session and reductions after a full treatment course [60], though results have been inconsistent across studies [61]. One possible interpretation of our findings is that the observed decrease in B-cell proportions reflects a broader shift towards immune system rebalancing or a dampened pro-inflammatory state, as also suggested by prior reports of reduced IL-6 and TNF-α levels during ECT [62]. These observations align with increasing evidence of immune-to-brain communication in psychiatric disorders [63] and the proposed role of immunological pathways in the mechanisms of ECT [64]. However, since our analyses rely on methylation-derived cell estimates, which are compositional by nature and cannot be interpreted in isolation, further studies combining epigenetic, cellular, and cytokine-based immune profiling are needed to better understand the interplay between immune modulation and clinical response.

Although this is the largest EWAS on ECT to date, it is still limited in its sample size. We identified DMPs and DMRs in relatively small cohorts, but larger cohorts would allow to identify more and might lead to better understanding of ECT mechanisms. Furthermore, there was a promising trend with MS, which in larger cohort could lead to MS being useful as biomarker for response prediction either alone or in combination with other markers (e.g. demographic, clinical, genetic) which would be highly relevant clinically.

Another important limitation is the homogeneity of the current cohort, as all patients included in this study are of European ancestry. Epigenetic profiles, including DNA methylation patterns, are known to vary significantly across ethnic groups due to genetic and environmental factors. Therefore, our findings may not be directly generalizable to patients from other ethnic backgrounds. Future studies involving multi-ethnic cohorts are necessary to determine whether the identified epigenetic markers have consistent predictive value across diverse populations, and to understand potential ethnic differences in epigenetic responses to ECT.

Combining clinical samples collected from different clinics poses the problem of comparison of cohorts that are also heterogenous in the clinical characterisation. Several studies have shown that some of these scales can be translated [29, 30]. While there might be some variations between the clinical phenotypes, identifying effects across cohorts should identify effects that are common to the core clinical outcomes (e.g. reduction in symptoms) and which might thus be more transferable across cohorts. Furthermore, we considered only the reduction of symptoms as the trait of interest and did not correct for diagnosis (i.e. bipolar disorder or MDD) which might affect the results and would need to be investigated.

Longitudinal analyses may help elucidate the mechanisms involved in ECTexp and ECTresp. The results from the meta-analyses were consistent with the single EWAS studies which found no significant associations with ECTexp or the interaction between ECTexp and ECTresp in either the Norwegian or German cohorts. The identification of longitudinal changes is sensitive to the time points at which the measurements are done. It is possible that some changes appeared and disappeared again before the measurements were taken, while other responses may be delayed. The lack of significant findings does not mean that there are no epigenetic changes involved with ECTexp. It could mean that the time points which were selected are not optimal, or it could indicate that larger samples are needed to detect this effect. Thus, further analyses increasing the number of sampling time points may help understand the dynamic of the changes identifying for example acute changes during treatment and long-lasting changes associated with long term remission.

It is expected that the direct effect of ECT involves the brain, and it may not be possible to obtain a detailed understanding of epigenetic mechanisms involved in ECTresp or ECTexp from blood. Physiological states relevant for response to ECT are likely to be reflected also in other tissues, including blood. Furthermore, DNAm has been shown to be correlated between brain and peripheral tissues [65–67]. Interestingly, while we identified changes associated with response in the blood which might be driven by individual specific traits (e.g. genetics) since they are present before treatment, we did not find changes associated with exposure to ECT. In a mouse model, Guo et al. [68] show rapid changes in DNA methylation in neurons after exposure to ECT which might be brain specific and can not be capture in the blood.

This study makes an important contribution to the field of epigenetics investigating antidepressant response. Although previous studies have shown diverging patterns in terms of which genes may be implicated in ECTresp or ECTexp, they all show some involvement of epigenetic mechanisms. This calls for larger studies in this field, including meta-analyses across all efforts and especially to progress towards the development of biomarkers for predicting response to ECT treatment which would be highly valuable for clinical decisions.

## Data availabilty

Methylation genotypes can be shared within a data transfer agreement for both samples, upon request of collaboration according to local ethical guidelines.

## Supplementary information

Supplementary Tables

Supplementary Figures
