# Supplementary material for: Epigenetic and blood markers associated with response to electroconvulsive therapy in patients with depressive disorders
Source: Transl Psychiatry. 2025 Dec 3;16:32. doi: 10.1038/s41398-025-03772-y (PMC12811393; doi:10.1038/s41398-025-03772-y)
Supplement: Supplementary file 2 — Supplementary Figures [file 41398_2025_3772_MOESM2_ESM.docx]

**SUPPLEMENTARY INFORMATION**

## Epigenetic and blood markers associated with response to electroconvulsive therapy in patients with depressive disorders

**Page 2 - Supplementary Figure 1**

Comparison of MADRS and HAMD21

**Page 3 - Supplementary Figure 2**

Estimated cell type proportions in non-responders, responders that did not remit, and remitters, before (baseline) and after treatment.

**Page 4 - Supplementary Figure 3**

Clusters of GO terms in the pathway analysis of DMRs associated with percent response.

**Page 5 - Supplementary Figure 4**

Clusters of GO terms in the pathway analysis of DMRs associated with remission.

**Page 6 - Supplementary Figure 5**

Clusters of GO terms in the pathway analysis of DMRs associated with delta score (meta-analysis).

**Page 7 - Supplementary Figure 6**

Clusters of GO terms in the pathway analysis of DMRs associated with binary response (meta-analysis).


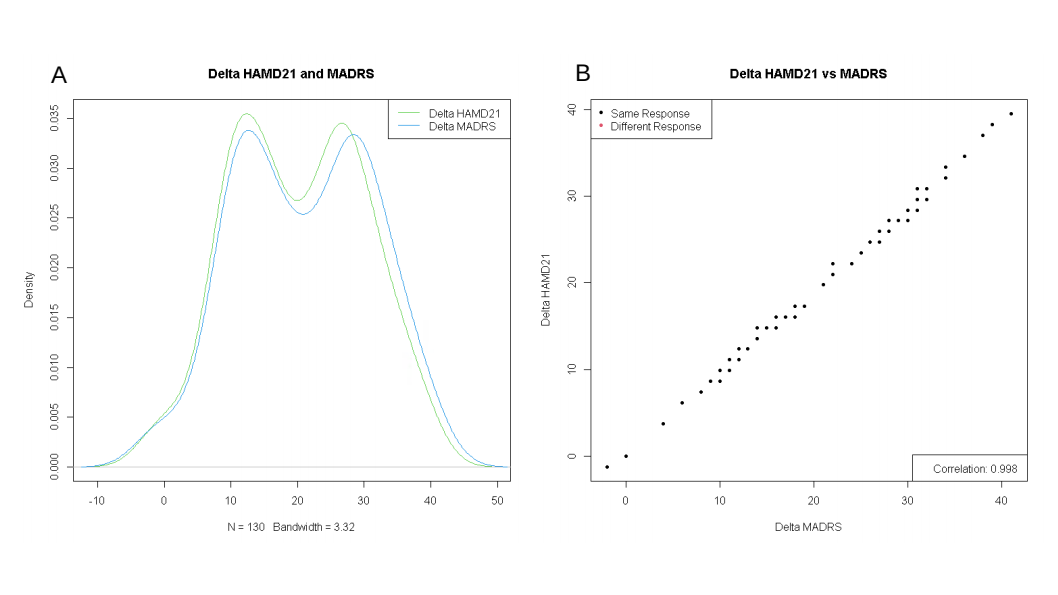
**Supplementary Figure 1** Comparison of the Delta MADRS scores and Delta HAMD21 scores for the Norwegian cohort. The MADRS were obtained from the clinical questionaire, while HAMD21 was obtained by converting MADRS to HAMD21. A) The distribution of the Delta MADRS and Delta HAMD21. B) Delta MADRS vs Delta HAMD21 and their correlation. All dots are black, showing that responder – non-responder groupings are the same using either MADRS or HAMD21.

**
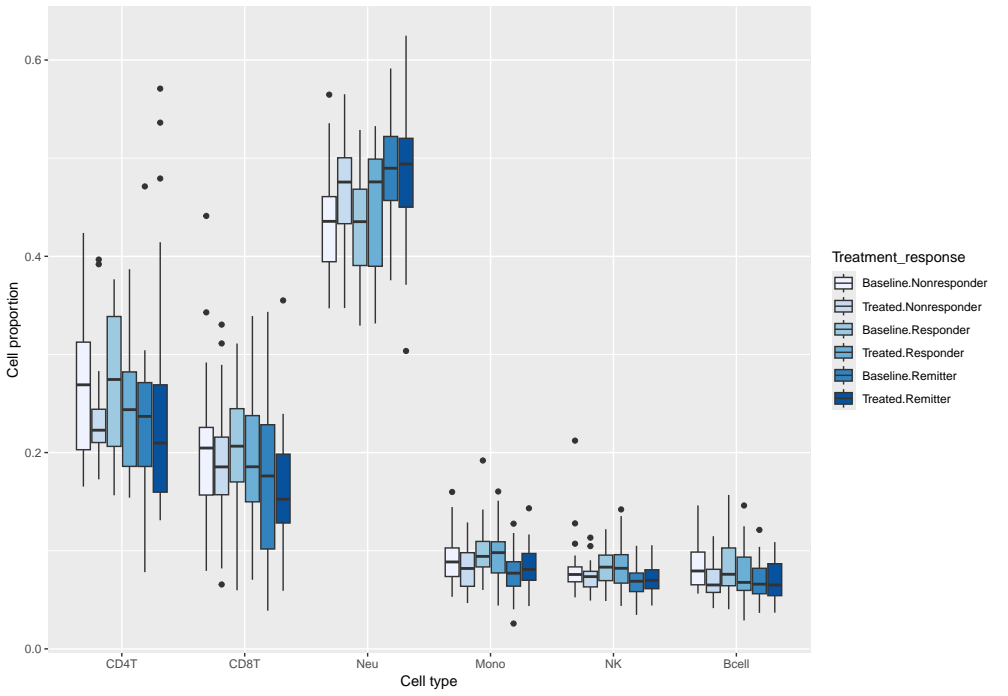
**

**Supplementary Figure 2** Estimated cell type proportions in non-responders, responders that did not remit, and remitters, before (baseline) and after treatment.


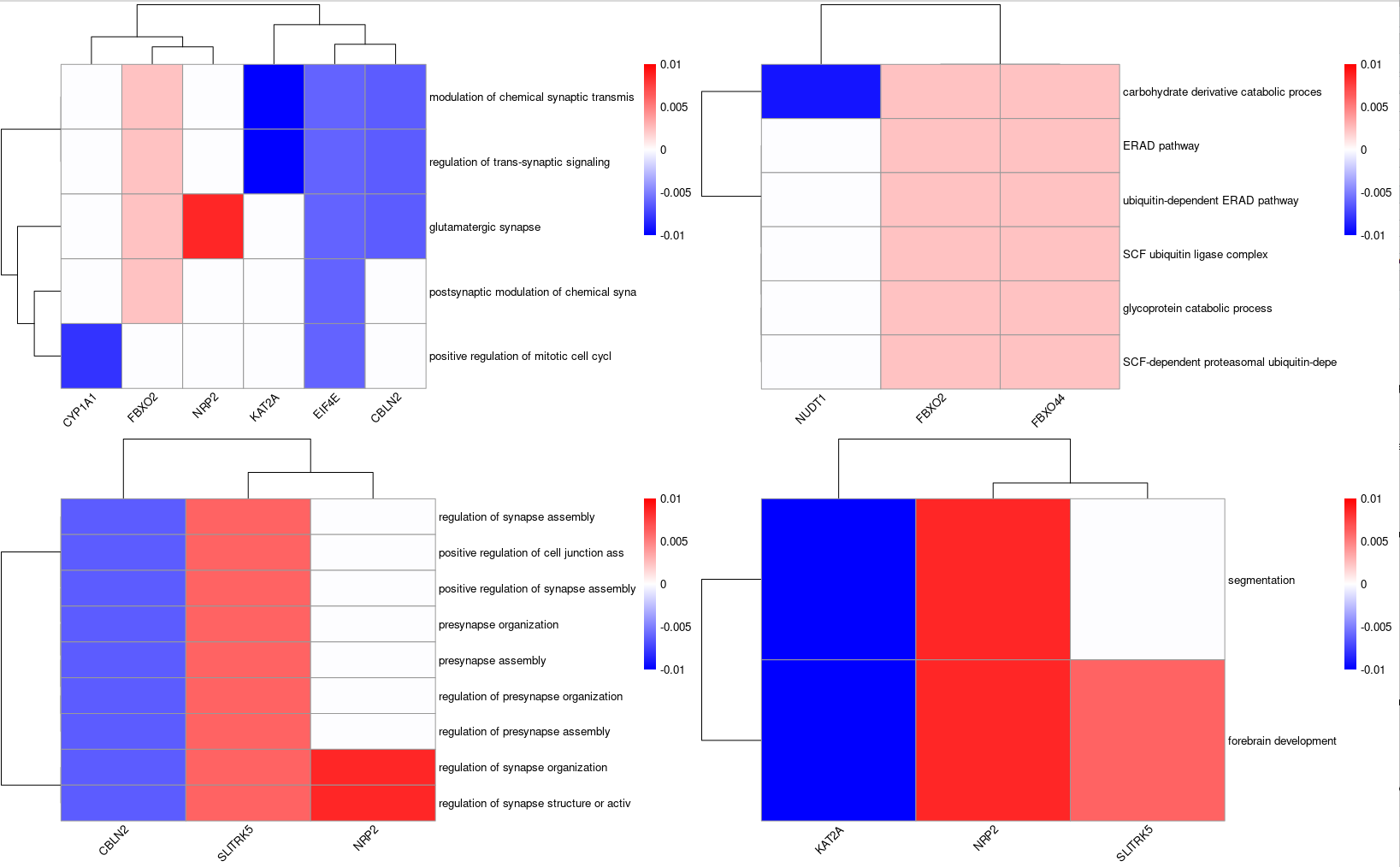


**Supplementary Figure 3** Clusters of gene ontology (GO) terms with a p-value < 0.01 in the pathway analysis of DMRs associated with percent response. The colours in the heatmap represent the effect size of the CpG with the lowest p-value within the DMR associated with a gene. Red represents a positive association between DNAm and response (i.e. higher DNAm is associated with better response), while blue represents a negative association between DNAm and response (i.e. lower DNAm is associated with better response).

**
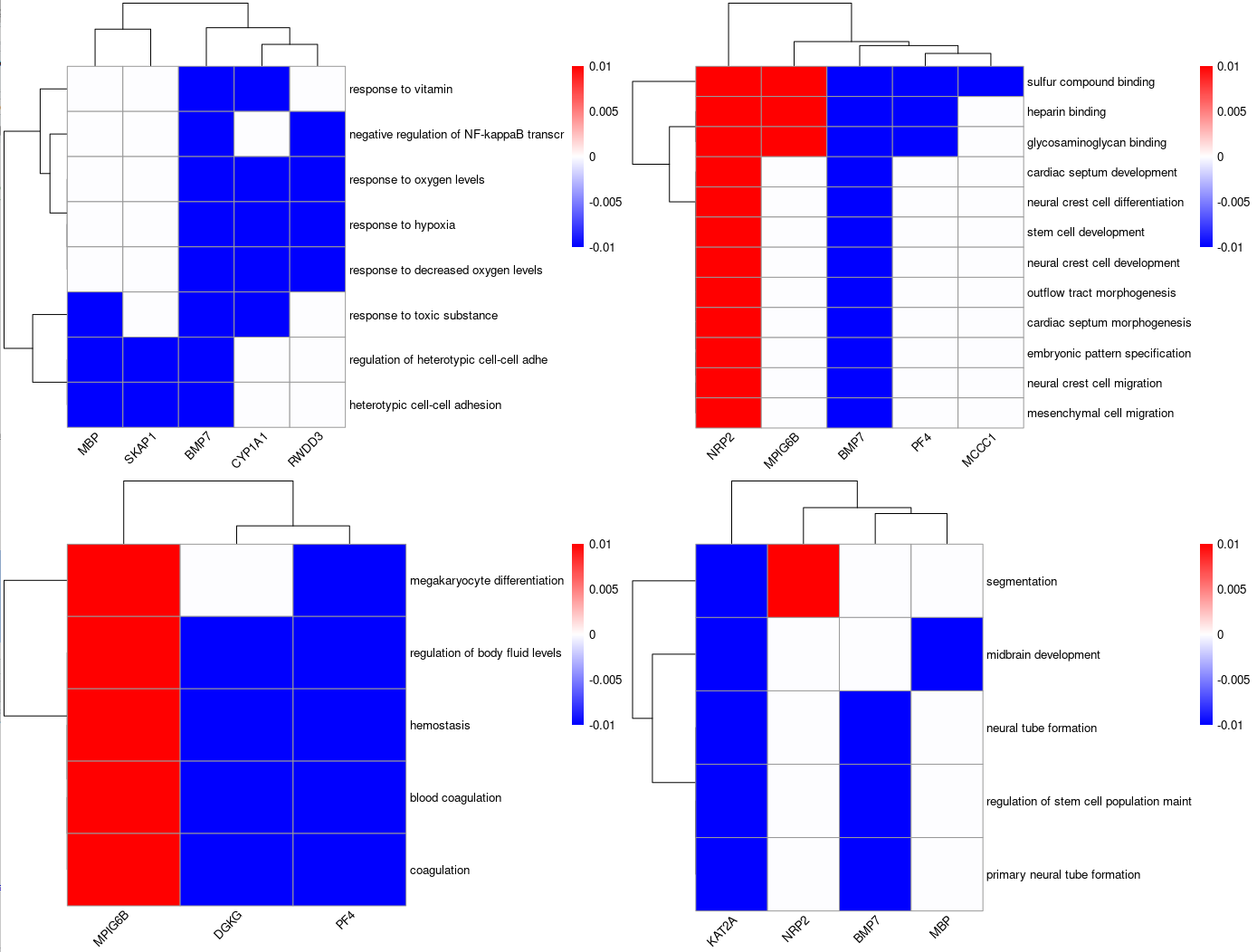
**

**Supplementary Figure 4** Clusters of gene ontology (GO) terms with a p-value < 0.01 in the pathway analysis of DMRs associated with remission. The colours in the heatmap represent the effect size of the CpG with the lowest p-value within the DMR associated with a gene. Red represents a positive association between DNAm and remission (i.e. remission is associated with higher DNAm), while blue represents a negative association between DNAm and remission (i.e. remission is associated with lower DNAm).

**
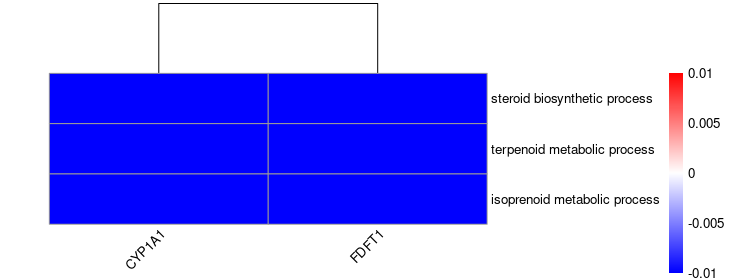
**

**Supplementary Figure 5** Clusters of gene ontology terms with a p-value < 0.01 in the pathway analysis of DMRs associated with delta score (meta-analysis). The colours in the heatmap represents the effect size of the CpG with the lowest p-value within the DMR associated with a gene. Red colour represents a positive association between DNAm and response (i.e. higher DNAm is associated with better response), while blue represents a negative association between DNAm and response (i.e. lower DNAm is associated with better response).


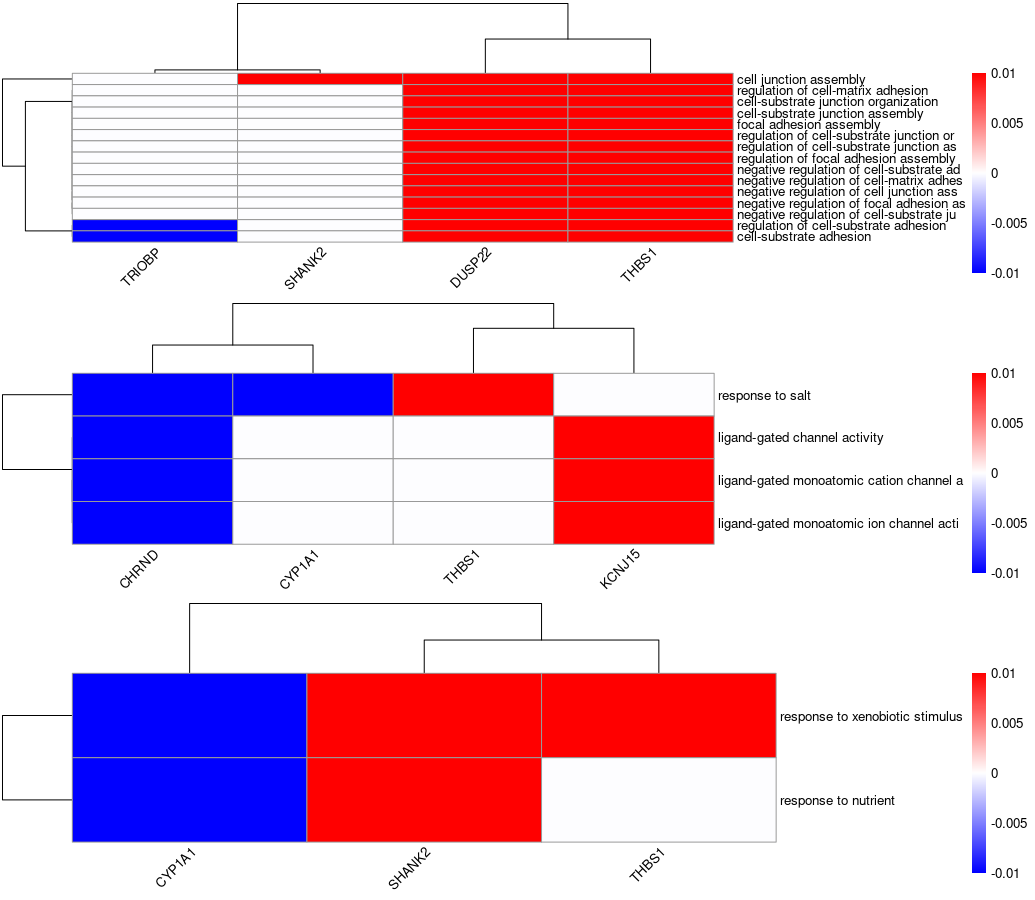


**Supplementary Figure 6** Clusters of gene ontology (GO) terms with a p-value < 0.01 in the pathway analysis of DMRs associated with binary response (meta-analysis). The colours in the heatmap represents the effect size of the CpG with the lowest p-value within the DMR associated with a gene. Red represents a positive association between DNAm and response (i.e. response is associated with higher DNAm), while blue represents a negative association between DNAm and response (i.e. response is associated with lower DNAm).
